# Current Advances in Developing New Antimicrobial Agents Against Non-Tuberculous *Mycobacterium*

**DOI:** 10.3390/antibiotics14121189

**Published:** 2025-11-21

**Authors:** Jane Cross, Nupur Gargate, Khondaker Miraz Rahman

**Affiliations:** Institute of Pharmaceutical Science, King’s College London, 150 Stamford Street, London SE1 9NH, UK

**Keywords:** nontuberculous mycobacteria, antimicrobial agents, antibiotic resistance, *Mycobacterium avium* complex, *Mycobacterium abscessus*, drug development, macrolide resistance, beta-lactams, oxazolidinones, fluoroquinolones

## Abstract

Non-tuberculous mycobacteria (NTM) comprise more than 190 species capable of causing severe pulmonary, lymphatic, cutaneous, and disseminated infections, particularly in immunocompromised populations. Over the past two decades, the global incidence of NTM infections has risen steadily, underscoring an urgent unmet medical need. Treatment remains highly challenging due to intrinsic antimicrobial resistance and the requirement for prolonged multidrug regimens that are often poorly tolerated and associated with unsatisfactory outcomes. At the same time, the development of novel therapies has lagged behind other disease areas, hindered by the high costs of antimicrobial drug discovery and the relatively low commercial return compared with treatments for chronic conditions. Over the past decade, discovery and development have diversified across novel small molecules, next-generation analogues of existing classes, and adjunctive or host-directed strategies. While most candidates remain preclinical, several agents have advanced clinically in other infections, including **gepotidacin** (topoisomerase inhibitor; FDA-approved 2025 for urinary tract infection (UTI)), sulbactam–**durlobactam** (DBO β-lactamase inhibitor; FDA-approved 2023 for *Acinetobacter baumannii* complex), and **contezolid**, supporting repurposing opportunities for NTM. Conversely, **SPR720** (gyrase B prodrug) was suspended after not meeting its Phase 2 endpoint in 2024, underscoring translational risk. Overall, the NTM pipeline is expanding, with near-term progress most likely from repurposed agents and optimised combinations, alongside earlier-stage candidates that target biofilms or resistance mechanisms. This review aims to provide a critical and up-to-date overview of emerging antimicrobial strategies against NTM, highlighting recent advances, translational challenges, and opportunities to accelerate the development of effective therapeutics.

## 1. Introduction

Non-tuberculous mycobacteria (NTM) comprise a diverse group of over 190 species and subspecies, widely distributed in the environment, particularly in water, soil, animals, and food sources. They can colonise mucosal surfaces, form biofilms, and persist intracellularly, making eradication with current antibiotics challenging [1]. NTMs are opportunistic pathogens capable of causing pulmonary, skin, and soft tissue infections, with pulmonary disease presenting as chronic cough, fever, fatigue, and weight loss [2]. Cutaneous and soft tissue manifestations include cellulitis, ulcers, and abscesses. The global incidence of NTM infections has risen in recent years, particularly among immunocompromised populations.

### 1.1. Types of NTM

Common types of NTM include *Mycobacterium avium* complex *(MAC)*, *Mycobacterium abscessus*, *Mycobacterium kansasii*, and *Mycobacterium smegmatis* The most prevalent of these is MAC, followed by *Mycobacterium abscessus* and *Mycobacterium kansasii* [1].

#### 1.1.1. *Mycobacterium avium* Complex (MAC)

MAC is the most common cause of NTM disease. These slow-growing organisms do not conform to traditional Gram-positive or Gram-negative classifications but are instead categorised as acid-fast due to their resistance to standard staining techniques. The group primarily includes *Mycobacterium avium* and *Mycobacterium intracellulare*. MAC infections are typically indolent but progressive, often requiring prolonged multidrug therapy, especially in individuals with chronic lung disease or HIV/AIDS [3].

#### 1.1.2. *Mycobacterium abscessus*

*Mycobacterium abscessus* is a rapidly growing, biofilm-forming, Gram-positive mycobacterium. Like all mycobacteria, it possesses a cell wall rich in mycolic acids, but its unique structural features contribute to substantial antibiotic resistance. It is also capable of intracellular growth within human macrophages, which adds to the complexity of treatment [4]. *Mycobacterium abscessus* is particularly problematic in patients with cystic fibrosis (CF) [5].

#### 1.1.3. *Mycobacterium kansasii*

*Mycobacterium kansasii* is a slow-growing, acid-fast mycobacterium associated with pulmonary infections that can mimic tuberculosis [6]. It may also cause lymphadenitis, skin and soft tissue infections, and disseminated disease in immunocompromised individuals [7]. Like MAC, it does not fit neatly into Gram-positive or Gram-negative categories.

#### 1.1.4. *Mycobacterium smegmatis*

*Mycobacterium smegmatis* is a non-pathogenic, acid-fast mycobacterium commonly found in soil and water. While it can occasionally cause skin infections, it is generally considered safe and is widely used in research as a surrogate model for pathogenic mycobacteria such as *Mycobacterium tuberculosis* [8]. Its genetic and structural similarities make it a valuable tool for early-stage drug development, where efficacy against *Mycobacterium smegmatis* may indicate potential against *Mycobacterium tuberculosis*.

### 1.2. Challenges in Developing New Antibacterials for NTM

NTM is particularly problematic for people with underlying issues that impact the immune system, such as cystic fibrosis, chronic obstructive pulmonary disorder (COPD), and HIV, with some strains such as *Mycobacterium abscessus* being particularly difficult to treat in these patient populations. The clinical management of NTM is complicated by these comorbidities. Furthermore, the burden on these patients can be high with the need to take multiple drugs for long periods, both orally and intravenously, with the potential for surgery as a last resort.

Another challenge in the development of new drugs for NTM is the lack of predictive animal models. Traditional mouse models often fail to sustain NTM infections long enough to evaluate drug efficacy meaningfully [9]. Zebrafish are another commonly used model due to their genetic tractability and physiological similarity to humans [10]. They are useful as embryos can be generated quickly and easily, and they are optically transparent so bacterial colonisation can be easily observed [10]. However, neither of these models can fully replicate the complexities of human biology, especially in individuals with comorbidities and compromised immune systems as seen in many NTM patients; therefore, their translatability is limited. Furthermore, many preclinical studies report results in relation to minimum inhibitory concentration (MIC); however, it remains challenging to translate MIC values into clinical efficacy for NTM. As a review by Griffiths and Winthrop notes, most NTM drugs show poor or inconsistent correlations between in vitro MICs and real-world treatment outcomes, making MICs an unreliable guide to predicting clinical response [11].

Research into new treatments is further constrained by the high cost of drug development and the relatively low profitability of antibiotics compared to treatments for chronic conditions, meaning that the return on investment is challenging. In a discussion paper on the growing crisis in antibiotic research and development, the Wellcome Trust reports that the cost of developing and bringing a new drug to market is GBP 1.9 billion, whereas peak global sales of a new antimicrobial agent are only GBP 260 million per year, meaning that return on investment is not attractive [12]. One reason for this is that new antimicrobials will be used as a last resort, once all other treatment options have failed, and therefore the market share is poor. Reimbursement can also be challenging when so many generic products are available, and these drugs tend to attract low prices [12].

Added to this, drug development is a long process with high attrition rates at each development stage. The estimated rate of conversion for a New Chemical Entity (NCE) in Phase 1 into an approved pharmaceutical product is ~10% [13]. These challenges have contributed to the significant medical need to identify and progress additional treatment options.

## 2. Epidemiology and Prevalence of NTM

The prevalence of NTM is challenging to identify due to it being difficult to diagnose by clinicians and not mandatory to report. However, in many countries, the prevalence of NTM has now exceeded that of *Mycobacterium tuberculosis* [14], with the number of cases per year in the USA believed to be between 13.9 and 48 per 100,000 people [1].

**Figure 1:**
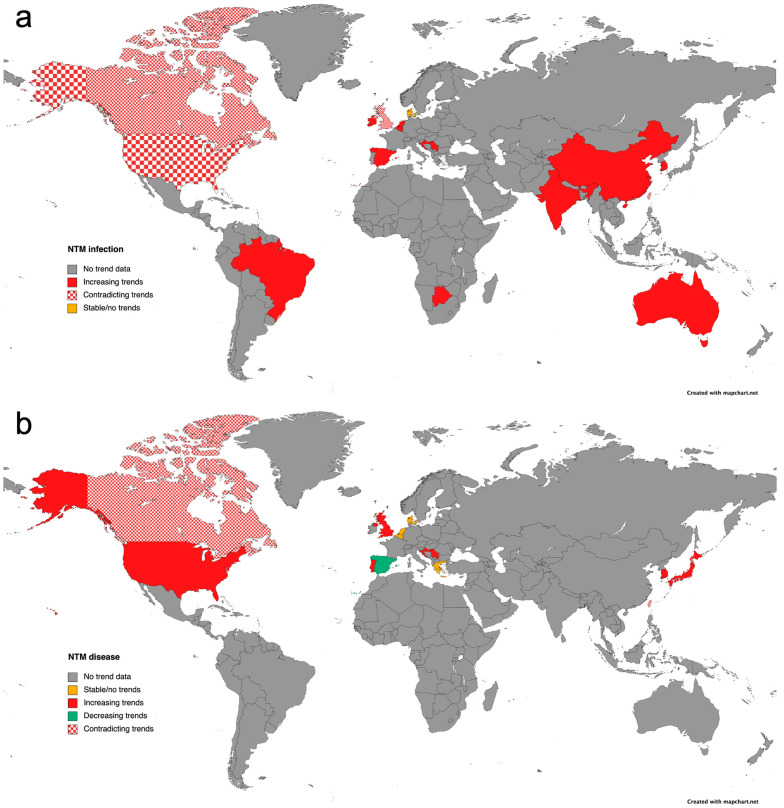
Global prevalence of NTM. (**a**) Prevalence of NTM infection worldwide. (**b**) Prevalence of NTM disease worldwide [15].

Figure 1a,b show the global prevalence of NTM pulmonary infection and NTM pulmonary disease, respectively [14]. As can be seen, where trends are available, the trend is increasing, with results showing an increase over time of 82% for NTM infection and 66% for NTM disease. However, there are many areas of grey where no trend data are available; as noted above, this could be due to the difficulties in diagnosing and the inconsistent global reporting of infection. Notably, for the UK, the rate of NTM infection appears to have a contradicting trend, and there is an increasing trend towards NTM disease.

Along with the global upward trend, there has also been an increase in prevalence of NTM in high-risk groups such as those with cystic fibrosis and COPD [15].

## 3. Resistance Mechanisms in NTM and Current Treatment Options

**Figure 2:**
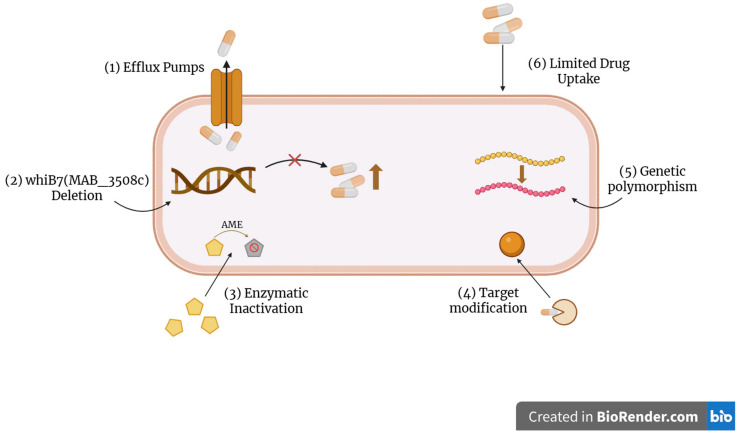
Common mechanisms of antibiotic resistance in NTM. Figure created using BioRender (https://www.biorender.com/).

NTM can be difficult to treat due to their resistance to many antibiotics. Both intrinsic and extrinsic resistance mechanisms prevail in NTM. Intrinsic resistance mechanisms include the presence of efflux pumps, reduced drug uptake in the bacteria, enzymatic inactivation of the antibiotic, and modification of bacterial targets. Additionally, resistance in NTM can be conferred by genetic polymorphism and the deletion of transcriptional regulators (Figure 2). Mycobacteria feature a thick, lipid-rich cell wall which acts as a physical barrier and reduces the uptake of antibiotics. This is complemented by the presence of efflux pumps, which actively expel antibiotics from the bacterial cell and reduce their effectiveness [16]. Intrinsic resistance is also conferred by enzymatic modification of antibiotics. A classic example of this is the modification of hydroxyl and amino groups in aminoglycoside antibiotics by aminoglycoside-modifying enzymes (AMEs), which renders aminoglycosides ineffective [16]. Furthermore, NTM species can modify the bacterial target sites that antibiotics act on, as seen commonly with macrolides. Erythromycin resistance methylase (*erm*) genes methylate 23S rRNA, the bacterial target of macrolides, and reduce macrolide binding affinity to the ribosome, conferring resistance [17]. Macrolide resistance is particularly concerning since these antibiotics are often the first-line treatment for NTM infections [18]. Moreover, recent studies have also shown that transcriptional regulators, particularly WhiB7, may participate in conferring resistance. When *M. abscesses* whiB7 (MAB_3508c) was deleted, increased susceptibility to macrolide, aminoglycoside, and tetracycline antibiotics was observed [19]. The impact of genetic polymorphisms in conserved drug target genes on antibiotic susceptibility in *M. abscessus* has also been studied [19]. A detailed discussion on the above can be found elsewhere [16,17,19].

Current treatment options are limited and require a combination of multiple antibiotics, and outcomes for patients are often poor. The first-line treatment is generally an antimicrobial macrolide such as clarithromycin (CAM) or azithromycin, as well as ethambutol and a rifamycin taken for up to eighteen months [20]. *Mycobacterium abscessus* is the most difficult-to-treat variant of the NTM species with a treatment success rate of only 45% [21]. It can lead to lung, skin, and soft tissue infections, as well as disseminated infections. Immunocompromised individuals, such as those with cystic fibrosis or HIV, are particularly susceptible to infection. There is no standard treatment for *Mycobacterium abscessus*; treatment consists of a multidrug regime preceded by in vitro macrolide susceptibility testing [22]. Furthermore, *Mycobacterium abscessus* can produce two different morphotypes: a rough morphotype which is more clinically prevalent, and a smooth morphotype that can form biofilms, making it even harder to treat [4].

MAC can cause infection in the respiratory tract and lymph nodes, as well as disseminated infections. The most common type of infection is pulmonary disease (NTM-PD). Like many forms of NTM, MAC is resistant to treatment with antibiotics [1]. The current recommendation for the treatment of MAC is antimicrobials, usually multidrug- and macrolide-based, given over many months [22].

## 4. Advances in Drug Discovery and Development for NTM

NTM is a significant and rising global health threat with cases increasing and antibiotic resistance posing a problem for treatment, particularly where comorbidities are present. Lack of investment from pharmaceutical companies due to the high attrition rate for new molecules, the challenges of clinical translatability, and the poor return on investment have added to the challenges with populating the drug development pipeline. This review will focus on current advancements in finding solutions to this public health threat.

### 4.1. New Small-Molecule Compounds

**Figure 3:**
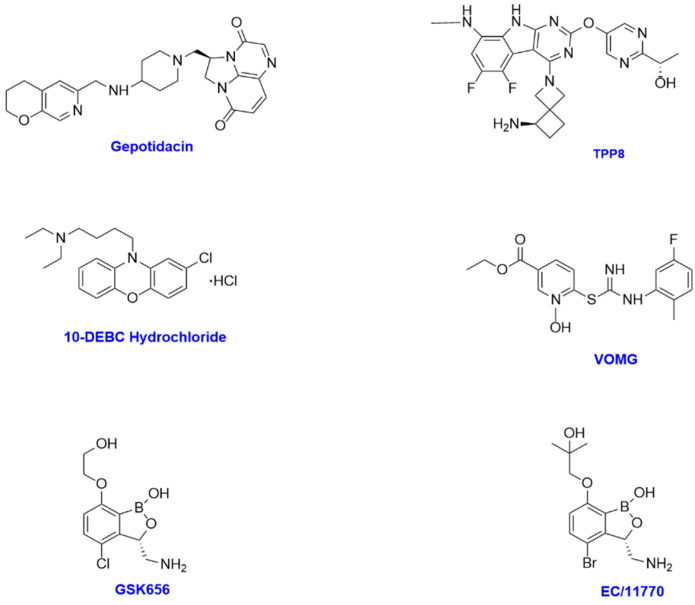
Chemical structures of some novel small-molecule compounds showing potential for NTM therapy.

Benzoxaboroles are boron heterocyclic compounds that represent a potential new drug class for the treatment of a range of pharmacological targets, including NTM (Figure 3) [23]. **EC/11770**, a benzoxaborole, is a novel leucyl-tRNA synthetase inhibitor that has shown efficacy against *Mycobacterium tuberculosis* and good anti-NTM activity in vitro, including against the difficult-to-treat *Mycobacterium abscessus* and the most prevalent NTM, MAC. **EC/11770** was shown to inhibit growth of *Mycobacterium abscessus* in a biofilm growth assay with MICs between 0.7 and 1.2μM, including an MIC of 0.7 μM for the commonly used ATCC 19977 reference strain and 0.3 μM for MAC (Table 1) [24]. Tackling biofilm formation is a key challenge in NTM therapy, and these results are encouraging. In a murine infection model, **EC/11770** produced a statistically significant 1.5 log reduction in lung CFU [24]. The compound also shows favourable physicochemical properties, including solubility of 349 μM, ChromLogD pH 7.4 of 0.97, and mouse clearance of 5 mL/min/kg, suggesting a likely BCS class of 1 and no anticipated formulation barriers.

Dong and colleagues tested **GSK656**, a 3-aminomethyl 4-halogen benzoxaborole, against 82 NTM isolates and demonstrated excellent activity against *Mycobacterium abscessus,* with all isolates showing MICs ≤ 25 mg/L. However, inherent resistance was noted for *Mycobacterium intracellulare* and MAC isolates, which displayed MICs > 8.0 mg/L [25]. In October 2023, GSK published encouraging results from a Phase 2 open-label clinical trial with **GSK656** for the treatment of tuberculosis [26]. This programme may accelerate development in NTM by leveraging the existing safety package.

**TPP8**, a tricyclic pyrrolopyrimidine (TPP) candidate, targets DNA gyrase and prevents DNA replication in a manner analogous to fluoroquinolones [27]. In vitro testing demonstrated potent activity against *Mycobacterium abscessus*, with MICs of 0.02–0.2 μM (Table 1). In an immunodeficient murine model, intraperitoneal dosing of 25 mg/kg reduced lung CFU approximately 20-fold, performing favourably compared to moxifloxacin and **SPR720** at the same dose. However, the lack of oral bioavailability may hinder its clinical development, potentially requiring reformulation or alternative delivery routes.

The novel compound **VOMG**, recently described as a cell division inhibitor, has demonstrated potent activity against *Mycobacterium abscessus* and other pathogens [28]. By targeting bacterial division, **VOMG** represents a distinct mechanism of action compared to traditional antibiotics. Early in vitro and preclinical results show significant bactericidal activity, positioning **VOMG** as a notable addition to the emerging pipeline of NTM therapeutics [28].

Another example of a novel mechanism is **10-DEBC hydrochloride**, which is believed to act as an Akt inhibitor. Akt plays a central role in cellular signalling, and inhibition interferes with pathways that allow cells to proliferate [22]. **10-DEBC** demonstrated activity against both clarithromycin-resistant and susceptible *Mycobacterium abscessus* strains, with MIC_90_ values of 2.38–4.77 μg/mL. It also showed activity against biofilms (MIC_90_ 50 μg/mL). To date, testing has been limited to in vitro work, and further studies are required to evaluate its in vivo potential and pharmacokinetic profile.

**Gepotidacin**, a first-in-class triazaacenaphthylene topoisomerase inhibitor, has recently been approved by the FDA for the treatment of urinary tract infection [29]. Against NTM isolates, **gepotidacin** was effective against ATCC 19977, with an MIC of 2 mg/L, comparing favourably to amikacin at 8 mg/L [30]. In a murine model of *M. fortuitum*, **gepotidacin** was more potent than amikacin at a 10-fold lower concentration. These findings suggest strong potential for repurposing to NTM indications, with the advantage of a robust safety data package already in place.

**Cyclophostins**, bicyclic compounds bearing a phosphonate moiety, have been investigated as inhibitors of serine hydrolases in mycobacteria, including *Mycobacterium abscessus* [31]. These compounds, and the closely related **cyclipostins**, display antimycobacterial activity both in extracellular cultures and within macrophages, with activity-based protein profiling suggesting targets among lipid metabolism and cell wall-associated enzymes. More recently, **cyclipostin/cyclophostin** analogues were shown to inhibit the erm(41) methyltransferase and thereby restore macrolide susceptibility in *Mycobacterium abscessus*, offering a potential route to overcoming inducible clarithromycin resistance [32]. Despite these promising findings, work on **cyclophostins** remains at the hit-to-lead stage, far earlier in the discovery pipeline compared with other compounds highlighted in this review.

Taken together, five candidates—**gepotidacin**, **TPP8**, **10-DEBC**, **GSK656**, and **EC/11770** (Figure 2)—stand out as promising additions to the sparse pipeline of novel agents for NTM. While **cyclophostins** remain at an early discovery stage, **gepotidacin** and **GSK656** are already in clinical development for other infections, providing an accelerated path forward for NTM indications. Among the preclinical candidates, **EC/11770** appears especially promising given its activity against both *Mycobacterium abscessus* and MAC in biofilm and murine infection models, coupled with excellent physicochemical properties. The recent addition of **VOMG** highlights the growing diversification of mechanistic approaches being explored for NTM, broadening the potential avenues for future therapy.

### 4.2. Scaffolds of Existing Drugs

**Figure 4:**
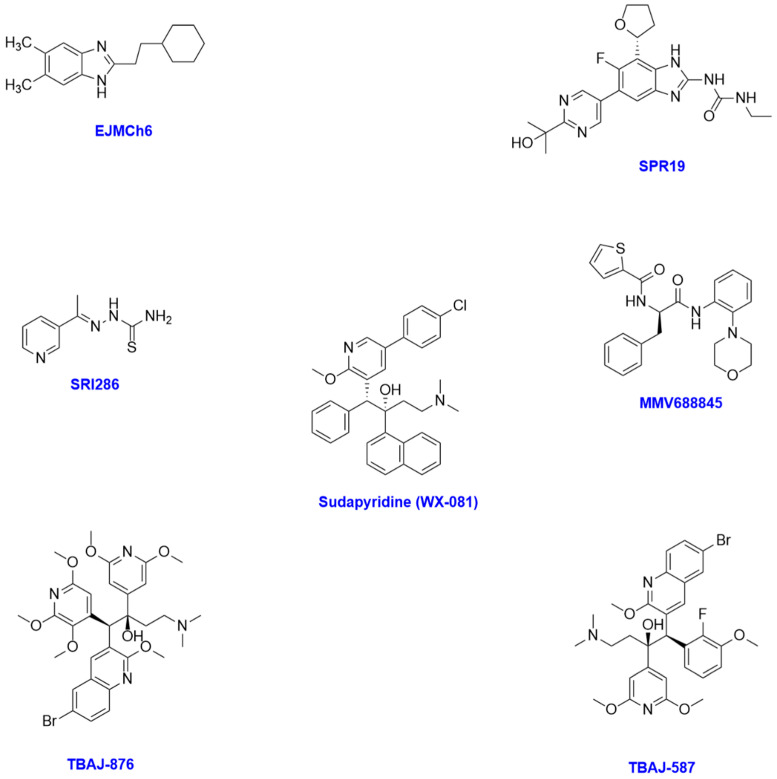
Chemical structures of some scaffolds repurposed from existing drug classes for potential treatment of NTM infections.

The following section describes a range of new molecules that are scaffolds of drugs (Figure 4) being used for the treatment of other indications, such as mefloquine, a traditionally antimalarial agent, and bedaquiline, used for the treatment of *Mycobacterium tuberculosis*.

Benzimidazoles’ primary mechanism of action in the context of mycobacterium is microtubule disruption by binding to beta-tubulin subunits. From the literature, two new molecules are under development as next-generation benzimidazoles—**EJMCh-6** and **SPR719/720**. **EJMCh-6** is a new benzimidazole which targets the MmpL3 transporter. MmpL3 targeting is seen as a promising target for the treatment of NTM. Drugs work by inhibiting this protein and thereby inhibiting its transporter function, leading to cell death. **EJMCh-6** was tested for the treatment of *Mycobacterium abscessus* in vitro using the CFU method, giving MICs of 2 μg/mL against a range of clinical isolates [33]. These assays were performed using well-characterised reference and mutant strains, including *Mycobacterium abscessus* CIP104536ᴛ smooth and rough variants, PIPD1-resistant mutants, and MmpL3 overexpression and point mutation strains, including A309P. **EJMCh-6** appears to be at the lead candidate phase, and no pharmacokinetic or in vivo data are reported, making it unclear how druggable the molecule is and hence appearing to be a long way from being a clinical candidate.

**SPR719** is a new benzimidazole that inhibits the ATPase activity of DNA gyrase B [34]. **SPR719** has demonstrated good in vitro activity against *Mycobacterium abscessus*, MAC, and *Mycobacterium kansasii*, with MICs ranging from 0.1 to 2.0 μg/mL [35]. For these MIC studies, **SPR719** was evaluated against a well-defined panel of laboratory and clinical NTM reference strains (Table 2). Furthermore, Pidot et al. investigated the in vitro activity of **SPR719** in several NTM isolates that primarily cause skin infections (*Mycobacterium ulcerans*, *Mycobacterium marinum,* and *Mycobacterium chimaera*), finding MICs in the range of 0.125–4 μg/mL [36]. These studies employed well-characterised panels of clinical and environmental reference strains.

**SPR720**, a prodrug that rapidly converts to **SPR719**, was being developed by Spero Therapeutics and successfully completed Phase 1 clinical trials, achieving a predicted therapeutic, once-daily oral dose in the range of 500–1000 mg [37], and progressed into Phase 2 studies. However, interim results released in 2024 indicated that the trial did not meet its primary efficacy endpoint, with bacterial clearance rates similar to background therapy. Spero has thus suspended further development on this compound [38,39]. While these results temper expectations for near-term clinical translation, the underlying potency of **SPR719** reinforces DNA gyrase B as a validated target for NTM drug discovery, and future structural analogues may overcome the limitations encountered with **SPR720**.

**844** is a novel piperidine-4-carboxamide (P4C) and DNA gyrase inhibitor originally identified through in silico screening, which showed efficacy against *Mycobacterium abscessus* with MICs between 6 and 14 μM (Table 2) [40]. Similar to the benzimidazole **SPR719**, it works by inhibiting DNA gyrase in NTM by binding and inhibiting DNA supercoiling, leading to bactericidal death [41]. Despite showing promising MICs, **844** did not perform well in pharmacokinetic assays, with rapid elimination after both oral and subcutaneous administration in the mouse. Furthermore, it had limited permeability and metabolic instability in the mouse model, although it was more stable in rabbit, monkey, and human plasma [40]. Nonetheless, these pharmacokinetic characteristics suggest that **844** would be challenging to formulate into a viable oral formulation for NTM.

Like **EJMCh-6** and the other benzimidazoles described above, **PIPD1** targets the mycolic acid transporter Mmlp3. **PIPD1** was tested against a range of clinical isolates of *Mycobacterium abscessus* with an MIC of 0.125 μg/mL reported (Table 2). Furthermore, it was found to perform well in infected zebrafish embryos, with a statistically significant reduction in deaths when treated with 3 μg/mL **PIPD1** compared to the untreated group [42]. During pharmacokinetic studies, **PIPD1** was found to have low relative bioavailability in the mouse, so further modification is envisaged to improve the potential of **PIPD1** before it becomes a clinical candidate [43].

**Gallium compounds** are a potential novel therapy against NTM. These compounds work by inhibiting Fe metabolism, disrupting iron-dependent metabolism pathways and therefore preventing bacterial proliferation. **Gallium nitrate (Ga(NO_3_)_3_)** and **Ga-protoporphyrin** showed potential as antimicrobial agents in reference and clinical strains of *Mycobacterium abscessus* [44]. This paper does not report on MICs in the same way as other papers, making it challenging to assess the comparability of these compounds. However, the paper did report that the findings were statistically significant. The team reported that **Ga(NO_3_)_3_** has been tested in a Phase 1 clinical study of CF patients being treated for *P. aeruginosa* infection, with no significant toxicity reported [44]. Furthermore, **gallium nitrate** is currently in a Phase 1b open-label trial (enrolment started in 2021) for the treatment of adults with CF who are colonised with NTM [45]. In the study, the patients are continuously infused with gallium nitrate over five days at a dose of 200 mg/m^2^/day. The study is estimated to be completed in 2025. **Ga-protoporphyrin**, has only been tested in vitro, and therefore the toxicity profile and in vivo efficacy are yet to be explored [44].

Bermudez and colleagues explored four active enantiomers of mefloquine against MAC in a mouse model of infection. They found that these enantiomers were not as effective as mefloquine, with MIC_50_ between 32 and 64 μg/mL vs. 16 μg/mL for mefloquine. However, they postulated that these enantiomers could still be a viable treatment option as the neurotoxic burden of mefloquine is known to be high, whereas an early evaluation of toxicity in embryonic rat cell neurons in vitro showed that these were ~47% less toxic than mefloquine [46]. In an earlier paper, Bermudez and colleagues looked at **SRI-286**, an experimental thiosemicarbazone with good efficacy of 2 μg/mL in vitro and in vivo against MAC. **SRI-286** was tested in combination with mefloquine and moxifloxacin for the treatment of MAC in mice, and it was found to be more effective than either treatment on its own [47]. However, the combination of mefloquine and moxifloxacin also shows good efficacy, which may call in to question the need to develop a novel drug when two marketed products work well in combination.

Following a library screen of molecules previously found to be active against *Mycobacterium tuberculosis*, Mann and colleagues identified and tested **MMV688845**, an RNA polymerase inhibitor, against a range of clinical isolates of *Mycobacterium abscessus* and found it to be broadly active, representing a promising starting point for a hit-to-lead discovery programme in this pathogen [48]. **MMV688845** displayed activity against multiple reference strains and clinical isolates of *Mycobacterium abscessus* (Table 2), with an MIC_90_ ranging from 4.5 to 10 µM. It was also evaluated in macrophage infection models, both alone and in combination with other antibiotics such as rifabutin and rifampicin, where additive effects were observed [48]. However, despite its in vitro potency, **MMV688845** demonstrated poor bioavailability in vivo, limiting its immediate potential as a direct clinical candidate. Structural optimisation strategies have therefore been suggested, with a particular focus on shielding the molecule’s amide bonds from enzymatic hydrolysis to improve pharmacokinetic properties [48,49].

**TBAJ-876** and **TBAJ-587** are next-generation diarylquinoline analogues of bedaquiline developed to reduce lipophilicity and cardiotoxicity while retaining or enhancing antimycobacterial activity. **TBAJ-876** has advanced furthest in development and is now in phase 2 clinical trials for *Mycobacterium tuberculosis* [50]. It demonstrates lower cardiotoxic potential, potent activity against *Mycobacterium abscessus* (MIC 0.48–1.05 μM in ATCC 19977 and clinical isolates), and additive or synergistic effects with standard NTM agents such as clarithromycin and cefoxitin [51].

**TBAJ-587** has also completed Phase 1 trials though no results have been published [52]. In a large study of 11 reference strains and 194 clinical isolates, it showed strong in vitro and in vivo activity against *Mycobacterium abscessus*, with MIC values significantly lower than those of bedaquiline, imipenem, and clarithromycin [53]. Importantly, Xu et al. demonstrated that **TBAJ-587** exhibited superior efficacy to bedaquiline in a murine model of tuberculosis carrying an Rv0678 resistance mutation, underscoring its potential to overcome resistance mechanisms that could also impact NTM therapy [54]. Although **TBAJ-876** currently leads in clinical progression, both **TBAJ-876** and **TBAJ-587** represent promising candidates to overcome the limitations of bedaquiline, with potential to strengthen therapeutic regimens for drug-resistant *Mycobacterium abscessus* and other NTM infections.

**Sudapyridine (WX-081)**, another next-generation diarylquinoline, has progressed in recent years with a growing body of preclinical and clinical data. Initial in vitro studies demonstrated potent activity against a wide range of NTM isolates, including *Mycobacterium abscessus*, with MIC values lower than those of bedaquiline [55]. This was followed by studies in a zebrafish infection model, where **WX-081** showed significant in vivo activity against *Mycobacterium abscessus* [56]. Most recently, Zheng and colleagues confirmed both in vitro and in vivo efficacy against multiple NTM species, reinforcing its potential as a clinical candidate [57]. After successfully completing a Phase II clinical trial, it is currently undergoing Phase III trials. While **WX-081** is further along the development pipeline than many experimental compounds, its clinical safety profile and comparative efficacy relative to **TBAJ-876** and **TBAJ-587** remain to be fully defined.

**Salicylanilides** are a class of compounds with potential therapeutic application in a range of disease areas, including the treatment of NTM. They demonstrated MIC values ranging from 0.125 to 8 μM against reference strains of MAC and reference and clinical strains of *Mycobacterium kansasii* [58]. Although these are strong MIC ranges, the team did not test **salicylanilides** against the most treatment-resistant strain of NTM-*Mycobacterium abscessus*, which appears to be a limitation of the study. This could be because, alongside NTM species, the study also assessed *Mycobacterium tuberculosis* and *Staphylococci*, and the team may be prioritising these disease areas over NTM. This study is still in the in vitro phase and has not progressed to any in vivo assessments of efficacy, positioning it in the early stages of the development cycle.

### 4.3. Other Antibiotics

**Figure 5:**
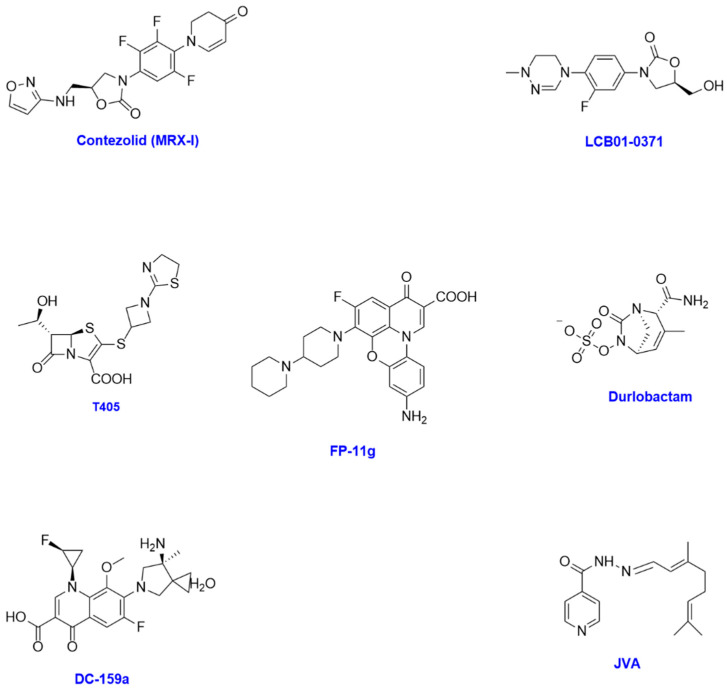
Chemical structures of some next-generation antibiotics with the potential for treating NTM.

One of the major focusses for new treatments is next-generation antibiotics. New antibiotics are a promising area of discovery, with many showing strong minimum inhibitory concentration levels against reference and clinical isolates of NTM. Additionally, many of the new antibiotics under review (Figure 5) are currently being progressed for the treatment of *Mycobacterium tuberculosis* or other bacterial infections and therefore have potentially a faster path to approval as repurposed drugs.

Guo and colleagues explored a novel oxazolidinone called **MRX-1 (contezolid)** against 12 reference strains and 194 clinical isolates of NTM. It was most effective against *Mycobacterium abscessus,* with MICs between 0.25 mg/L and 64 mg/L (Table 3) [59]. **MRX-1** is currently undergoing clinical trials for the treatment of pulmonary tuberculosis and diabetic foot disease [60,61] and has already completed a trial for Gram-positive bacterial skin infections [62].

Kim et al. [63] tested the activity of **LCB01-0371** against *Mycobacterium abscessus* in vitro as well as in a murine model of infection, where it was found to have comparable efficacy to linezolid with a reduction in CFU in lungs to 3.7 log^10^. However, it did not perform as well in the spleen and liver [63]. LCB01-0371 has successfully completed Phase 1 clinical trials for safety and tolerability [64] and has also completed Phase 2 clinical trials for the study of pulmonary tuberculosis at doses ranging from 800 mg to 1200 mg per day [65]. However, as it has not shown to be as efficacious as other treatment options available for NTM (such as linezolid) in animal models, it is questionable whether it should progress further if demonstrable differentiation (such as improvement of side effects) is not seen.

Madani et al. examined the antibacterial activity of 19 **oxadiazolone-core derivatives** to determine their antibacterial activity against both rough and smooth variants of *Mycobacterium abscessus* [66]. These **OX derivatives** had previously shown promise against *Mycobacterium tuberculosis* with a potentially diverse mechanism of action, as they worked both extracellularly on bacterial growth and intracellularly on infected macrophages, while showing low toxicity against host macrophages. These new **oxadiazole-core derivatives** showed MIC_50_ ranges between 33–>120 μM against a combination of rough and smooth types of *Mycobacterium abscessus* (Table 3) [66]. These MICs are not seen as efficacious enough to warrant further investigation as potential drug candidates, but are useful as a focus for understanding the pathogenesis of NTM and potential treatment options.

With two new oxazolidines (**MRX-1** and **LCB01-0371**) already in the clinic for tuberculosis, this is a particularly promising area of development with potential for a fast regulatory approval pathway as repurposed drugs, provided regulatory approval in *Mycobacterium tuberculosis* is obtained.

Batchelder and colleagues tested **T405**, a new beta-lactam antibiotic of the penem subclass, against a panel of *Mycobacterium abscessus*. It showed an MIC of 2 mg/mL against reference strain ATCC 19988, compared to existing beta-lactams which showed MIC_90_ of 16–32 μg/mL for imipenem and 32–64 μg/mL for cefoxitin [67]. Furthermore, **T405** showed MICs between 1 and 8 μg/mL in the 21 clinical isolates tested, demonstrating that it has good antimicrobial activity against a diverse range of isolates (Table 3). Additionally, the team tested **T405** in a mouse pharmacokinetic model and found it to have a favourable half-life of 0.82 h when dosed subcutaneously, which they hypothesised is due to high protein binding [67]. No physicochemical data are provided to enable an assessment of **T405**’s potential to be formulated into an oral dosage form, and no in vivo efficacy is provided in the paper; therefore, it is difficult to assess its feasibility as a drug candidate.

Diazabicyclooctanes (DBOs) are a class of beta-lactam inhibitors. **Durlobactam** is a DBO being investigated for its ability to augment the activity of beta-lactams by targeting beta-lactamases that confer resistance to beta-lactam antibiotics [68]. **Durlobactam** was used in combination with amoxicillin, imipenem, and cefuroxime to assess whether the combination increased the MIC relative to the beta-lactams on their own [68]. Results were best when using a combination of IMI-DUR-AMOX and CXM-DUR-AMOX, with ≤0.06–2 μg/mL and ≤0.06–1 μg/mL, respectively. **Durlobactam** appears to inhibit beta-lactamase enzymes and prevent them from hydrolysing the beta-lactam ring, therefore enabling them to work more effectively [68]. Additionally, they appear to interrupt peptidoglycan biosynthesis. In 2023, **durlobactam** received FDA approval for the treatment of *Acinetobacter baumannii* in combination with sulbactam [69], having successfully completed a Phase 3 clinical trial [70]. Therefore, it is in a strong position for a fast path to clinical trials and repurposing as a treatment option for NTM.

**DC-159a** was explored in vitro against a range of NTM isolates, with MICs reported in the range of 0.03–32 μg/mL, including an MIC in *Mycobacterium abscessus* with a range of 4–32 μg/mL [71]. Although the data in the study are in vitro, the team had completed a pharmacokinetic study in a monkey model administering an oral dose of 5 mg/kg. The team reported that **DC-159a** achieved a higher peak concentration than another formulation, but it is unclear from the study whether it was within the target range [71].

A fluroquinophenoxazine compound (**FP-11g**) was tested against clinical isolates of *Mycobacterium smegmatis* and *Mycobacterium abscessus and* found to inhibit growth in both strains. For *Mycobacterium smegmatis*, the MIC was 0.31 µM, and in *Mycobacterium abscessus* it was 50 µM. However, the figure for *Mycobacterium abscessus* is quite high, particularly when compared to the reference strains of clarithromycin at 1–2 µM and ciprofloxacin at 38 µM [72]. The team appear to suggest that **FP-11g** would be useful as a drug to treat patients who have *Mycobacterium tuberculosis* and another strain of NTM, rather than as a treatment for NTM in isolation.

Pflegr et al. synthesised and tested **isoniazid scaffolds** in clinical isolates of *Mycobacterium kansasii* and MAC. The research mainly focused on *Mycobacterium tuberculosis,* and the team designed compounds based on the **isoniazid scaffold** and assessed the structural-activity relationship to design an improved compound. The results showed some level of MIC in both strains (8–250 m/M for *Mycobacterium kansasii* and 250–500 m/M for MAC) [73]. However, the research appears to be in the very early hit-to-lead phase and is focusing primarily on *Mycobacterium tuberculosis,* and is therefore unlikely to have a positive impact on advancing treatments for NTM in the short term. Furthermore, the MICs reported are quite high compared to other promising candidates in this review.

**JVA** is an isoniazid analogue which demonstrated an MIC of 320 µM against MAC [74]. However, it was not shown to be as efficient as clarithromycin at reducing bacterial growth. Additionally, the team assessed the ability of **JVA** to reduce the growth of MAC in mouse-derived macrophages and found that in this assay it was more effective than both clarithromycin and isoniazid. This team hypothesised that the increased activity in macrophages is due to the presence of neutral forms of the compound at pH 6.5–7.4, which enables **JVA** to cross macrophage membranes [74]. With mixed results in early models, it is difficult to assess whether this will become a viable candidate for progression.

Although isoniazid is a first-line treatment for MAC, these new analogues have also focused on MAC, limiting their potential as the highest unmet medical need is in *Mycobacterium abscessus*. They are also earlier in development than other new antibiotics and are not in the clinic for other antimicrobial diseases and are therefore a long way from being a treatment option for patients.

In summary, analogues of existing antibiotics are a promising area of discovery. The most notable analogue is **MRX-1**, which is currently in clinical trials for pulmonary tuberculosis and shows good efficacy against reference strains of *Mycobacterium abscessus,* with MICs between 0.25 μg/L and 64 μg/L, as well as an improved safety profile over linezolid [59]. **Durlobactam**, approved for the treatment of *Acinetobacter baumannii* in combination with sulbactam, is another promising candidate [69]. Many of the studies are still in the preclinical stage; however, others have progressed through to the clinical stage for the study of pulmonary tuberculosis and other bacterial infections. If approved in other indications, they could be repurposed for the treatment of NTM. This is particularly true for the oxazolidine class of antibiotics, where two analogues are in clinical trials for tuberculosis.

## 5. Other Investigational Approaches

### 5.1. Peptide-Based Therapies

As well as small molecules, research is ongoing in a range of other areas including peptides, adjunctive therapies such as efflux inhibitors, drugs targeting biofilm formation, and fungal metabolites (Table 4).

Peptides are short chains of amino acids; therapeutic peptides represent a unique class of drugs and have been a focus for drug discovery since the approval of insulin as a therapeutic peptide over 100 years ago. The past ten years have also seen advances in manufacturing and analytical analysis in peptide drug discovery and development [75]. However, peptide development remains challenging, as many peptides exhibit undesirable properties as drugs; notably, many are inherently unstable [76]. In addition, their susceptibility to proteolytic degradation and rapid clearance often results in poor in vivo exposure, limiting therapeutic effectiveness. Furthermore, challenges related to delivery, manufacturing cost, and optimisation of membrane penetration continue to hinder the translation of peptide-based candidates for NTM therapy. [76]

Da Silva et al. [77] tested six antimicrobial peptides that had previously shown efficacy against *Mycobacterium tuberculosis* and *Mycobacterium smegmatis* against clinical isolates of *Mycobacterium abscessus*. The peptides showed MICs between 1.6 and >50 μg/mL, with AP1 being the most promising, with an MIC range of 1.5–3.1 μg/mL against well-characterised strains of NTM and MIC of 1.5–6.2 μg/mL against a further 25 clinical isolates of *Mycobacterium abscessus* (Table 4) [77]. Although promising, this is in the very early stage of development, having only been tested in vitro.

Sudadech et al. [78] tested thirteen novel AMPs in both a drug-resistant *Mycobacterium abscessus* isolate and a clinical isolate. Four out of the thirteen tested showed promising MICs, with values between 200 and 400 μg/mL against the Mab ATCC19977 strain (Table 4). These candidates were then tested in clinical isolates of *Mycobacterium abscessus*. Of the candidates tested, the AMPs that had been modified by the truncation of amino acid sequences, as opposed to the parent AMPs, showed better efficacy. The authors hypothesised that this is due to increased hydrophobicity leading to better interaction with the cell surface [78]. The research determined that the novel AMPs were most effective in combination with clarithromycin, where synergy was demonstrated (FICI 0.02–0.41), supporting their potential role as adjunctive therapies [78]. This was promising, as AMPs do carry a burden of toxicity, which is reduced if the dosing regimen is lower.

Cationic host defence peptides (CHDPs) are a subclass of oligopeptides which are being explored for their ability to impact the immune system. Rao et al. investigated the effectiveness of **NZX**, a CHDP originating from the fungus *Pseudoplectania nigrella*, in MAC and *Mycobacterium abscessus* [79]. The paper focused mainly on **NZX**’s efficacy in *Mycobacterium tuberculosis* models; however, an early assessment of the efficacy of 15 clinical isolates of NTM was completed, with **NZX** showing potent activity on both fast- and slow-growing bacteria (Table 4). The paper reports that a higher concentration of **NZX** is required for more treatment-resistant strains of NTM, such as *Mycobacterium abscessus* (12.5–25 mg/L) [79].

Since 2023, however, additional advances have been reported. **Arenicin-derived peptides** have been evaluated against *Mycobacterium abscessus*, with **Ar-1** demonstrating potent activity, minimal cytotoxicity, and no evidence of resistance development [80]. In parallel, **IAMP29**, a viral-derived immunomodulatory peptide, was shown to enhance macrophage killing of rapidly growing NTMs via NLRP3 inflammasome activation, suggesting a novel host-directed mechanism of action [81]. Beyond individual candidates, technological advances are accelerating peptide discovery; AI-driven design platforms such as AMP-Designer, as well as de novo computational pipelines, have rapidly produced peptides with improved stability, low toxicity, and demonstrated in vivo efficacy in bacterial lung infection models [82]. Together, these findings indicate that while peptide therapeutics for NTM remain preclinical, there is clear momentum in both mechanistic diversity and development tools that may shorten the path to clinical evaluation.

In summary, peptide therapeutics for NTM remain at a preclinical stage, with no candidates yet in clinical trials, but ongoing work highlights both direct antimicrobial activity and synergy with existing antibiotics as promising avenues for further development.

### 5.2. Adjunctive Therapies

Of the NTM species, MAC in particular has a high intrinsic resistance to multiple antibiotics due to drug efflux mediated by efflux pumps and a decreased permeability of cell walls. Therefore, a key target for drug development is in developing a suitable efflux pump inhibitor to be used alone or in conjunction with other antimicrobials to enable the antibiotic to remain in the cells and reach effective concentrations within the bacteria [83]. During a medicinal chemistry programme, Felicetti and colleagues identified **16a** as a promising candidate for further development and found that modifying the positions on C6 and C7 of 3-Phenylquinolone efflux pump inhibitors enabled the development of a molecule with an MIC of 128 μg/mL in MAC (Table 5) [83]. This programme is in the very early stages of development, with a relatively high MIC compared to other studies in this review.

The development of efflux inhibitors as adjunctive or primary-use pharmaceuticals is an interesting area of focus for antimicrobial drug development. The mechanism of action is clear and proven in in vitro models. However, these developments are still all in the in vitro stage, and Felicetti et al. report that other efflux inhibitors induced toxicity in in vivo models [83]. This is consistent with other preclinical data on efflux pump inhibitors, which have not progressed towards clinical development due to toxicity concerns. This is a reminder that drugs that look promising at the preclinical phase of development are not always able to progress into the clinic and beyond.

Biofilm formation is one of the ways in which NTM maintains its resistance to antibiotics; therefore, disruption of biofilm formation is a focus for drug development in this area. **RP557** is an antimicrobial peptide investigated for its ability to disrupt biofilm formation in M. *abscessus* [84]. Using the ATCC19977 strain, **RP557** reduced biofilm integrity and increased killing compared to an untreated control. Furthermore, it enhanced the activity of clarithromycin, amikacin, cefoxitin, and imipenem in combination. However, the reported value (16 µg/mL) refers to its biofilm activity rather than planktonic MIC, and **RP557** has only been tested in a single strain. Further work in clinical isolates and in vivo models is needed before it can be considered viable for development.

Another adjunctive therapy covered by the review is **NUNL02**, which is a derivative of tetrahydropyridine explored in combination with known antimicrobial agents to see if the addition of **NUNL02** improved the efficacy of these drugs against *Mycobacterium abscessus* [85]. The study found that the addition of **NUNL02** increased the MIC of the amikacin 16-fold and ciprofloxacin 4-fold [85]. **NUNL02** was only tested in vitro, with no in vivo studies described, suggesting this is at a very early stage of development.

In Asian countries such as Japan, China, and Korea, the antimicrobial properties of fungi- and plant-derived compounds have long been recognised. Millar and colleagues isolated 23 macrofungi belonging to the phylum *Basidiomycota* native to the UK and tested them against clinical isolates of *Mycobacterium abscessus*. All but one species inhibited both clinical and reference isolates, with a mean zone of inhibition of 8.7 mm [86]. However, the use of inhibition zones rather than MIC values makes comparison with other antimicrobials challenging. The authors suggested that activity may stem from phenolic acid production as a defensive mechanism in soil. This was the first assessment of these fungi against NTM and remains at an early stage of discovery; the mechanism of action and activity in animal models require further study.

More recently, **isoegomaketone**, a natural product from *Perilla frutescens*, has been identified as a potential inhibitor of *Mycobacterium abscessus* [87]. **Isoegomaketone** showed bactericidal activity, disruption of cell membrane integrity, and possible synergy with clinically used antibiotics, while exhibiting low cytotoxicity in mammalian cells. Although still preclinical, these findings support **isoegomaketone** as a candidate for further evaluation in NTM drug development.

In summary, there are a range of promising areas of development that fall outside of the classic small-molecule category, including peptides and adjunctive therapies such as efflux and biofilm inhibitors. As multidrug regimens are standard in the treatment of NTM, adjunctive therapies are an attractive option for further development, and **RP557** illustrates how biofilm-targeting peptides may enhance the efficacy of existing antibiotics, though further validation is required [81].

### 5.3. Alternative and Supportive Therapies

Other options for treatment include surgery, phage therapy, and gut microbe remodelling. These options will briefly be reviewed and summarised below (Table 6).

Most treatment options explored in this review are at the early phase of development and have centred around pharmaceutical options. However, one paper explored an early pulmonary resection surgery for MAC, which achieved a 100% success rate. In the study, 22 patients who were treatment-resistant to multiple antibiotics received either a lobectomy (n = 15), partial lung resection (n = 6), or a segmentectomy (n = 4). All patients were free of MAC sputum four months post-surgery [88]. The authors of the report recommend early surgical intervention before pulmonary lesions become too difficult to resect. There may be some resistance to surgery as a primary standard of care, as it is very invasive, risking complications and damage to surrounding tissue. Operating on already very sick patients is risky and may require long recovery times. Furthermore, it is limited in scope, meaning that all the infection may not be removed or may have spread to other parts of the body, requiring further surgery or risk of reinfection at the original site. Surgery is also much more labour-intensive and requires more resources than pharmaceutical interventions.

Phage therapy utilises bacteriophages to treat bacterial infections and is recognised as an alternative to conventional antibiotics. There are challenges with phage therapy as a treatment option, including the lack of phages available for NTM [89]. A paper by Dedrick and colleagues reviewed 20 patients treated with phage therapy in a clinical setting. Of those treated, 11 had favourable clinical or microbiological responses to the treatment with limited side effects. This is a promising treatment, as unlike a lot of the therapies in this review, it is available and has proven efficacy in patients rather than in animal models of infection. However, the response rate of 11 out of 20 is relatively low for such a labour-intensive and complex intervention. Like antibiotics, there is also a risk of phage resistance over time.

One paper explored the role that L-arginine can play in boosting immune defences against NTM. Patients with NTM were found to be deficient in L-arginine [90]. In the study, mice were administered arginine through oral administration, which boosted the gut microbiota composition with *Bifidobacterium* species. This resulted in a boost to pulmonary immune defence against NTM in mice treated for antibiotic resistance. Additionally, faecal microbiota transplantation also resulted in increased protection against NTM. These results suggest that gut microbe remodelling could be a promising area for further research [90]. However, there are challenges with this approach, as it is not easily reproducible because each microbiota is different. Additionally, it may result in gut microbiota changes that could increase susceptibility to other forms of infection. In summary, there are still a lot of unknowns with this novel approach.

5-aminolevulinic acid photodynamic therapy (ALA_PDT) is a medical treatment that selectively targets and destroys diseased cells. Wang et al. found that ALA_PDT can kill *Mycobacterium abscessus* in infections by promoting ferroptosis-like cell death [75]. This is the only study included in the review that focusses primarily on skin rather than lung infections. The study was in vitro only, and while suggesting that ALA_PDT may have a potentiating effect on antibiotics, it was acknowledged that a significant amount of research is required before this becomes a viable treatment option [75].

## 6. Future Perspectives

Most candidate therapies for NTM remain at the preclinical stage and, therefore, a long way from clinical use. The discontinuation of **SPR720** after not meeting its Phase 2 endpoint in 2024 illustrates the difficulty of translating promising early results into patient benefit. Nevertheless, there are reasons for cautious optimism. Several compounds have advanced in other disease areas and could be repurposed for NTM, which may shorten development timelines. Examples include oxazolidinones such as **contezolid**, next-generation diarylquinolines such as **TBAJ-587**, which completed Phase 1 dosing in healthy volunteers, and topoisomerase inhibitors such as **gepotidacin**, which received FDA approval in 2025 for urinary tract infection. **Durlobactam**, approved in 2023 for multidrug-resistant *Acinetobacter baumannii* in combination with sulbactam, demonstrates the clinical feasibility of this class, raising the possibility of applications in NTM. Adjunctive approaches, including **gallium nitrate**, biofilm-disrupting peptides, and host-directed therapies, add further diversity to the pipeline.

AI is increasingly being applied to accelerate antimicrobial discovery, offering promising opportunities for NTM. A review of the literature indicates that most AI developments to date have focused on improving diagnostics. Advances in genomic technologies, including whole-genome sequencing (WGS), have already improved species identification and resistance prediction, and recent work demonstrates that AI tools can streamline analysis, enabling more rapid and accurate diagnostic interpretation [91]. Machine learning approaches have enabled the rapid design and screening of antimicrobial peptides, improving hit rates, reducing toxicity, and demonstrating efficacy in preclinical models [92]. Both discriminative and generative models are being used to mine antimicrobial peptide databases and generate novel peptide libraries with enhanced antimicrobial properties [92]. In parallel, the lack of effective therapies, particularly against *Mycobacterium abscessus*, has stimulated the use of computational methods to accelerate early-stage compound discovery, although the authors note significant challenges in applying these tools to NTM drug discovery [93]. A key limitation is the lack of publicly available, good-quality data, both positive and negative, associated with chemical scaffolds with NTM activity, which affects the performance and reliability of AI-directed antimicrobial drug discovery. A concerted effort is therefore needed to improve AI models and ensure that training datasets more accurately represent real-world chemical and biological diversity. Overall, these AI-enabled tools have the potential to significantly shorten hit-to-lead timelines and optimise preclinical resource allocation, although they remain at an early stage and their clinical translation remains to be fully realised.

In recent years, regulatory agencies have taken steps to help address this unmet medical need. For instance, the FDA introduced the Qualified Infectious Diseases Product Designation (QIDPD), whereby drugs being developed as antimicrobials qualify for several incentives such as fast-track designation, priority review, and a five-year extension of exclusivity [94]. Researchers could make use of these incentives to accelerate drug development to address this urgent unmet need.

Taken together, the near-term therapeutic horizon is likely to be shaped by repurposed compounds and optimised drug combinations, while novel scaffolds, adjunctive strategies, and AI-enabled platforms will require further validation in clinically relevant NTM models. Although significant challenges remain, the broadening of the discovery pipeline over the past decade is encouraging and suggests that new therapeutic options for NTM, while not imminent, are becoming increasingly feasible.

## Figures and Tables

**Table 1:** Overview of novel small-molecule antibacterial compounds and their development stages.

Compound	Bacterial Strain(s)	MIC	Bacterial Target and Mechanism of Action	Development Status
**EC/11770**	*M. abscessus* Bamboo (subsp. *abscessus*) *M. abscessus* subsp. *Abscessus* (no. of strains = 7) *M. abscessus* subsp. *Massiliense* (no. of strains = 2) *M. abscessus* subsp. *Bolletii* (no. of strains = 3) *M. avium* 11 (subsp. *hominissuis*)) *M. intracellulare* (no. of strains = 1) *M. chimaera* (no. of strains = 1)	0.7–1.2 μM 0.33–0.93 μM 0.71–0.95 μM 0.48–1.3 μM 4.0 μM 0.37 μM 1.7 μM	Leucyl-tRNA synthetase inhibitor, interfering with bacterial protein biosynthesis	Preclinical stage
**GSK656**	*M. intercellulare* (no. of strains = 30) *M. avium* (no. of strains = 16) *M. abscessus* (no. of strains = 36)	>0.8 mg/L >0.8 mg/L 0.016–0.25 mg/L	Leucyl-tRNA synthetase inhibitor, interfering with bacterial protein biosynthesis	Phase II clinical trials
**TPP8**	*M. abscessus* subsp. *Abscessus* (no. of strains = 7) *M. abscessus* subsp. *Bolletii* (no. of strains = 3) *M. abscessus* subsp. *Massiliense* (no. of strains = 2)	0.02–0.2 μM 0.04–0.2 μM 0.1–0.2 μM	DNA gyrase, causing bacterial DNA damage	Preclinical stage
**VOMG**	*M. abscessus* subsp. *Abscessus* (no. of strains = 8) *M. abscessus* subsp. *Bolletii* (no. of strains = 1) *M. abscessus* subsp. *Massiliense* (no. of strains = 1) *M. avium* (no. of strains = 5) *M. bovis* (no. of strains = 5) *M. smegmatis* (no. of strains = 5)	0.25–0.5 µg/mL 0.5 µg/mL 0.5 µg/mL 0.5 µg/mL 0.0625 µg/mL 1 µg/mL	FtsZ enzyme, interfering with bacterial cell division	Preclinical stage
**10-DEBC hydrochloride**	*M. abscessus* (no. of strains = 9)	2.38–4.77 µg/mL	Akt inhibitor; unknown mechanism of action	Lead optimisation/preclinical stage
**Gepotidacin**	*M. fortuitum* ATCC 6841 *M. chelonae* ATCC 35752 *M. abscessus* ATCC 19977 *M. avium* ATCC 19698 *M. gordonae* ATCC 14470 *M. nonchromogenicum* ATCC 19530 *M. kansasii* ATCC 12478 *M. intracellulare* ATCC 13950	2 mg/L 2 mg/L 2 mg/L 16 mg/L 16 mg/L 32 mg/L 32 mg/L 16 mg/L	Triazaacenapthylene topoisomerase inhibitor, inhibiting bacterial type II topoisomerase and interfering with bacterial DNA replication	Preclinical stage
**CyC_17_**	*M. smegmatis* mc^2^155 *M. abscessus* CIP 104536^T^ *M. marinum* *M. bovis* BCG *M. abscessus* (no. of strains = 10) *M. massiliense* (no. of strains = 4) *M. bolletii* (no. of strains = 2) *M. chelonae* (no. of strains = 10)	0.81 µg/mL 0.18 µg/mL 0.74 µg/mL 0.58 µg/mL 10 µg/mL 10 µg/mL <2 µg/mL 40 µg/mL	Serine/cysteine hydrolase inhibitors, impairing bacterial lipid metabolism and cell wall assembly	Hit-to-lead identification

**Table 2:** Current progress of existing drug scaffolds repurposed or optimised for antibacterial activity.

Compound	Bacterial Strain(s)	MIC	Bacterial Target and Mechanism of Action	Development Status
**EJMCh-6**	*M. abscessus* (no. of strains = 12) *M. massiliense* (no. of strains = 12) *M. bolletii* (no. of strains = 9)	0.031–0.5 µg/mL 0.062–0.5 µg/mL 0.062–1 µg/mL	MmpL3 transporter, blocking cell wall mycolylation	Hit-to-lead identification/lead optimisation stage
**SPR719**	*M. avium* complex M. *avium* M. *intracellulare* *M*. *chimaera* MAC-X *M. abscessus* *M. abscessus* subspecies *abscessus* *M. abscessus* subspecies *massiliense* *M. abscessus/massiliense* hybrid *M. kansasii* *M. chelonae* *M. fortuitum* *M. immunogenum* *M. mucogenicum* *M. marinum* *M. simiae* M. *xenopi* *M*. *ulcerans*	0.002–4 µg/mL 0.23–2 µg/mL 0.12–2 µg/mL <0.03–2 µg/mL 0.12–1 µg/mL 0.03–>32 µg/mL 0.25–8 µg/mL 0.12–4 µg/mL 0.06–2 µg/mL 0.002–0.25 µg/mL 2–4 µg/mL 0.06–1 µg/mL 4–8 µg/mL 0.015–0.25 µg/mL 0.12–1 µg/mL 0.5–8 µg/mL 0.06–0.5 µg/mL 0.125–0.25 µg/mL	ATPase activity of DNA gyrase B, inhibiting bacterial DNA replication	Phase I clinical trials
**SPR20**	N/A	N/A	ATPase activity of DNA gyrase B, inhibiting bacterial DNA replication	Phase I clinical trials
**844**	*M. abscessus ATCC 19977* *M. abscessus* clinical isolates (no. of strains = 7) *M. bolletii* CCUG 50184T *M. bolletii* clinical isolates (no. of strains = 2) *M. massiliense* CCUG 48898T *M. massiliense* clinical isolates (no. of strains = 1)	8 μM 6.3–12 μM 14 μM 6.3 μM 14 μM 12.5 μM	DNA gyrase inhibitor, causing bacterial DNA damage	Lead optimisation/preclinical stage
**PIPD1**	*M. abscessus* (no. of strains = 12) *M. massiliense* (no. of strains = 12) *M. bolletii* (no. of strains = 8)	0.125 0.125 0.125	MmpL3 transporter, blocking cell wall mycolylation	Lead optimisation/preclinical stage
**Ga(NO_3_)_3_ & Ga-protoporphyrin**	N/A	N/A	Interference with bacterial iron metabolism, causing metabolic dysfunction	Phase I clinical trials
**SRI286**	MAC	2 μg/mL	Mycolic acid synthesis inhibitor, disrupting function and integrity of bacterial cell wall	Preclinical stage
**MMV688845**	*M. abscessus* subsp. *abscessus* ATCC 19977 *M. abscessus* clinical isolates (no. of strains = 7) *M. abscessus* subsp. *bolletii* CCUG 50184T *M. bolletii* clinical isolates (no. of strains = 2) *M. abscessus* subsp. *massiliense* CCUG 48898T *M. massiliense* clinical isolates (no. of strains = 1)	7.5 μM 5.4–8.4 μM 10 μM 4.5–6.9 μM 10 μM 8.4 μM	RNA polymerase inhibitor, exhibiting bactericidal activity	Lead optimisation/preclinical stage
**TBAJ-876**	*M. abscessus* subsp. *abscessus* ATCC 19977 *M. abscessus* clinical isolates (no. of strains = 6) *M. abscessus* subsp. *bolletii* CCUG 50184-T *M. bolletii* clinical isolates (no. of strains = 2) *M. abscessus* subsp. *massiliense* CCUG 48898-T *M. massiliense* clinical isolates (no. of strains = 1)	0.48 μM 0.14–0.46 μM 0.53 μM 0.30–0.45 μM 0.42 μM 0.30 μM	F-ATP synthase inhibitor, preventing ATP synthesis and thus bactericidal activity	Phase II clinical trials
**TBAJ-587**	*M. abscessus* ATCC 19977 *M. abscessus* clinical isolates (no. of strains = 148) *M. massiliense* CIP 108297 *M. massiliense* clinical isolates (no. of strains = 46) *M. smegmatis* ATCC 607 *M. fortuitum* ATCC 35855 *M. peregrinum* ATCC 700686 *M. avium* ATCC 25291 *M. intracellulare* ATCC 13950 *M. kansasii* ATCC 12478 *M. gordonae* ATCC 14470 *M. szulgai* ATCC 35799 *M. scrofulaceum* ATCC 19981	0.031 mg/L 0.0625 mg/L 0.031 mg/L 0.0625 mg/L 0.004 mg/L 0.008 mg/L ≤0.002 mg/L ≤0.002 mg/L ≤0.002 mg/L ≤0.002 mg/L ≤0.002 mg/L ≤0.002 mg/L 0.008 mg/L	Inhibitor of F-ATP synthase c-chain, exhibiting bactericidal activity	Phase I clinical trials
**Sudapyridine (WX-081)**	Rapidly growing *Mycobacterial* species (no of strains = 26) Slowly growing *Mycobacterial* species (no of strains = 24)	0.0078–0.5 μg/mL 0.0039–>2 μg/mL	ATP synthase inhibitor, preventing ATP production required for cellular activities	Phase III clinical trials
**Salicylanilide**	*M. avium 330/8* *M. kansaii 235/80* *M. kansasii 6509/96*	8 μM 1 μM 2 μM	Multiple mechanisms of action, inhibiting mycobacterial enzymes, regulatory systems, and impairing bacterial energy production	Hit-to-lead identification stage

**Table 3:** Summary of next-generation antibiotics under development for NTM treatment.

Compound	Bacterial Strain(s)	MIC	Bacterial Target and Mechanism of Action	Development Status
**Contezolid**	*M. abscessus* subsp. *abscessus* (ATCC 19977) *M. abscessus* subsp. *massiliense* (CIP108297) *Mycobacterium fortuitum* (ATCC 6841) *Mycobacterium smegmatis* (ATCC 19420) *Mycobacterium peregrinum* (ATCC 700686) *M. avium* (ATCC 25291) *M. intracellulare* (ATCC 13950) *Mycobacterium kansasii* (ATCC 12478) *Mycobacterium gordonae* (ATCC 14470) *Mycobacterium scrofulaceum* (ATCC 19981) *Mycobacterium marinum* (ATCC 927) *Mycobacterium xenopi* (ATCC 19250) *M. abscessus* subsp. *abscessus* (no. of strains = 148) *M. abscessus* subsp. *massiliense* (no. of strains = 46)	16 mg/L 16 mg/L 8 mg/L 1 mg/L 1 mg/L 32 mg/L 64 mg/L 1 mg/L 2 mg/L 1 mg/L 4 mg/L 1 mg/L 0.5–64 mg/L 0.25–64 mg/L	Bacterial protein synthesis inhibitor, interfering with bacterial growth and replication	Phase III clinical trials
**LCB01-0371**	*M. abscessus* ATCC 19977 *M. abscessus* clinical isolates (no. of strains = 8)	1.2 μg/mL 0.7–22.3 μg/mL	Bacterial protein synthesis inhibitor, interfering with bacterial growth and replication	Phase II clinical trials
**Oxadiazolone derivatives**	*M. abscessus* S-variant *M. abscessus* R-variant	3.9–>200 μM 7.4–>200 μM	Inhibits multiple bacterial enzymes, interfering with lipid metabolism and cell wall biosynthesis	Hit-to-lead identification stage
**T405**	*M. abscessus* ATCC 19977 *M. abscessus* clinical isolates (no. of strains = 20)	2 μg/mL 1–8 μg/mL	Inhibits penicillin-binding proteins and l,d-transpeptidases, inhibiting cell wall synthesis	Lead optimisation/preclinical stage
**Durlobactam**	*M. abscessus* ATCC 19977	2–8 μg/mL	Beta-lactamase inhibitor, improves beta-lactam activity and inhibits cell wall synthesis	Phase III clinical trials
**DC-159a**	*M. kansasii* (no. of strains = 22) *M. avium* (no. of strains = 33) *M. intracellulare* (no. of strains = 17) *M. fortuitum* (no. of strains = 10) *M. chelonae* (no. of strains = 10) *M. abscessus* (no. of strains = 12)	0.03–025 μg/mL 0.25–8 μg/mL 0.25–8 μg/mL 0.03–0.25 μg/mL 4–16 μg/mL 4–32 μg/mL	DNA gyrase, causing bacterial DNA damage and resulting in bactericidal effect	Lead optimisation/preclinical stage
**FP-11g**	*M*. *smegmatis* mc^2^155 *M*. *abscessus*	0.31 μM 50 μM	Bacterial topoisomerase and DNA gyrase, exhibiting bactericidal effect	Lead optimisation stage
**Isoniazid derivatives**	*M. avium* 330/88 *M. kansasii* 6509/96 *M. kansasii* 235/80	250–1000 μM 2–1000 μM 8–>250 μM	InhA enzyme inhibitor, inhibiting mycolic acid production and thus bacterial cell wall biosynthesis	Hit-to-lead identification stage
**JVA**	*M. avium* 2447	320 μM	Isoniazid derivative, which gets hydrolysed to isoniazid, inhibiting bacterial cell wall biosynthesis	Lead optimisation/preclinical stage

**Table 4:** Overview of antibacterial peptide therapies under development for NTM infections.

Peptide Name	Bacterial Strain(s)	MIC	Development Status
AMP1-AMP-6 AMP1 AMP2	*M. abscessus* ATCC 19977 *M. abscessus* subsp. *massiliense* MAB_062600_1635 *M. abscessus* subsp. *massiliense* MAB_030804_1651 *M. abscessus* subsp. *massiliense* MAB_010708_1655 *M. abscessus* clinical isolates (no. of strains = 25) *M. abscessus* clinical isolates (no. of strains = 25) *M. abscessus* clinical isolates (no. of strains = 25) *M. abscessus* clinical isolates (no. of strains = 25)	3.1–>50 μg/mL 1.6–>50 μg/mL 1.6–>50 μg/mL 1.6–>50 μg/mL 1.5–6.2 μg/mL >50 μg/mL 1.5–6.2 μg/mL >50 μg/mL	Hit-to-lead identification stage
S61, S62, S63 KLK1 S61 S62 S63 KLK1	*M. abscessus* ATCC 19977 *M. abscessus* ATCC 19977 *M. abscessus* clinical isolates (no. of strains = 16) *M. abscessus* clinical isolates (no. of strains = 16) *M. abscessus* clinical isolates (no. of strains = 16) *M. abscessus* clinical isolates (no. of strains = 16)	200 μg/mL 400 μg/mL 6.25–>400 μg/mL 12.5–>400 μg/mL 6.25–>400 μg/mL 25–>400 μg/mL	Hit-to-lead identification stage
NZX	*M. abscessus* (no. of strains = 3) *M. abscessus* subsp *abscessus* (no. of strains = 3) *M. abscessus* subsp *boletti* (no. of strains = 3) *M. gordonae* (no. of strains = 3) *M. xenopi* (no. of strains = 3) *M. kansasii* (no. of strains = 3) *M. lentiflavum* (no. of strains = 3) *M. avium* (no. of strains = 3) *M. shimodeii* (no. of strains = 3) *M. szulgai* (no. of strains = 3) *M. chimaera* (no. of strains = 3) *M. scrofulaceum* (no. of strains = 3) *M. intracellulare* (no. of strains = 3) *M. marinum* (no. of strains = 3) *M. chelonae* (no. of strains = 3)	12.5–25 mg/L 6.3–25 mg/L 3.2–25 mg/L 0.4–12.5 mg/L 0.4–0.8 mg/L 1.6–6.3 mg/L 3.2–25 mg/L 1.6–3.2 mg/L 0.4–3.2 mg/L 12.5–25 mg/L 0.4–1.6 mg/L 0.8–1.2 mg/L 0.4–3.2 mg/L 6.3–25 mg/L 0.4–3.2 mg/L	Preclinical stage
Arenicin peptides * Ar-1 Ar-1-Abu Ar-2 Ar-2-Abu Ar-3 Ar-3-Abu Ar-3(3–20) Ar-3(7–16)	*M. abscessus* CIP 104536^T^ *M. abscessus* CIP 104536^T^ *M. abscessus* CIP 104536^T^ *M. abscessus* CIP 104536^T^ *M. abscessus* CIP 104536^T^ *M. abscessus* CIP 104536^T^ *M. abscessus* CIP 104536^T^ *M. abscessus* CIP 104536^T^	11.4/17.5–11.6/20.2 μM >100 μM 19.8/29.3–53.1/>100 μM 89.3/ > 100–>100 μM 5.3/12.2–44.7/> 100 μM >100 μM 48.3/> 100–77.8/ > 100 μM 17.2/21.3–24.6/ > 100 μM	Lead optimisation stage

* All MIC values are expressed as peptide minimal concentration leading to 50% and 90% inhibition of in vitro growth, respectively (MIC50/MIC90), in S- and R-variant of M. abscessus CIP 104536^T^.

**Table 5:** Summary of adjunctive therapies for NTM infections and their development status.

Antimicrobial Agent	Bacterial Strain(s)	MIC	Bacterial Target and Mechanism of Action	Development Status
16a	*M. smegmatis* mc^2^155 *M. avium*	32 μg/mL 128 μg/mL	Bacterial efflux pump inhibitor, boosting the activity of co-administered antibiotics	Hit-to-lead identification/lead optimisation
RP557	N/A	N/A	Inhibitor of bacterial biofilm formation	Lead optimisation/preclinical stage
NUNLO2	*M. abscessus* subsp. *abscessus* ATCC 19977 *M. abscessus* subsp. *abscessus* (AT 07) *M. abscessus* subsp. *bolletii* (AT 46) *M. abscessus* subsp. *bolletii* (AT 52)	200 μg/mL 100 μg/mL 100 μg/mL 50 μg/mL	Bacterial efflux pump inhibitor, boosting the activity of co-administered antibiotics	Lead optimisation
Basidiomycota macrofungi	N/A	N/A	Unknown bacterial target and mechanism of action	Hit discovery/exploratory phase
Isoegomaketone	*M. abscessus* ATCC 19977 *M. abscessus* clinical isolates (no. of strains = 8)	128 μg/mL 32–128 μg/mL	Exact mechanism of action is unknown, but bactericidal and anti-biofilm activity, and disruption of cell membrane is observed	Lead optimisation/preclinical stage

**Table 6:** An overview of alternative and supportive therapies for NTM treatment.

Intervention	Description	Challenges
Pulmonary resection surgery	Subjects resistant to multiple antibiotics received either a lobectomy, partial lung resection, or a segmentectomy, and were free of MAC sputum four months post-surgery.	Potential for resistance emerging to surgery and risk in operating on very sick people. Labour-intensive and requires more resources.
Phage therapy	A total of 11 out of 20 subjects in the study demonstrated favourable clinical responses with limited side effects.	Lack of phages available for NTM treatment and risk for emergence of resistance. Potential translational and regulatory approval challenges.
Gut microbe remodelling	Oral administration of arginine in mice boosted pulmonary immune defence against NTM and faecal microbiota transplants showed increased protective host defence.	Complexity and differences in microbiota limits reproducibility of this intervention.
ALA_PDT	Promotes ferroptosis-like death of *M. abscessus* and antibiotic sterilisation through oxidative stress.	Further animal and clinical experiments are required to define exact molecular basis and clinical utility.

## Data Availability

This is a review article, and no primary data has been generated as part of this study.

## Abbreviations

The following abbreviations are used in this manuscript:

AI	Artificial intelligence
AMOX	Amoxicillin
AMP(s)	Antimicrobial peptide(s)
ATCC	American Type Culture Collection
BCS	Biopharmaceutics classification system
CF	Cystic fibrosis
CFU	Colony-forming units
CHDP	Cationic host defence peptide
COPD	Chronic obstructive pulmonary disease
CXM	Cefuroxime
DBO(s)	Diazabicyclooctane(s)
DNA	Deoxyribonucleic acid
DUR	Durlobactam
FDA	U.S. Food and Drug Administration
FICI	Fractional inhibitory concentration index
GSK	GlaxoSmithKline
HIV	Human immunodeficiency virus
IMI	Imipenem
MAC	*Mycobacterium avium* complex
Mab	*Mycobacterium abscessus*
MIC	Minimum inhibitory concentration
MIC_50_	MIC required to inhibit 50% of organisms
MIC_90_	MIC required to inhibit 90% of organisms
Mmpl3	Mycobacterial membrane protein large 3
NCE	New chemical entity
NLRP3	NLR family pyrin domain containing 3
NTM	Non-tuberculous mycobacteria
NTM-PD	Non-tuberculous mycobacterial pulmonary disease
P4C	Piperidine-4-carboxamide
QIDPD	Qualified Infectious Diseases Product Designation
WGS	Whole-genome sequencing
